# Changes in Cognition and Mortality in Relation to Exercise in Late Life: A Population Based Study

**DOI:** 10.1371/journal.pone.0003124

**Published:** 2008-09-01

**Authors:** Laura E. Middleton, Arnold Mitnitski, Nader Fallah, Susan A. Kirkland, Kenneth Rockwood

**Affiliations:** 1 Geriatric Medicine Research Unit, Centre for Health Care of the Elderly, Halifax, Nova Scotia, Canada; 2 Department of Community Health and Epidemiology, Dalhousie University, Halifax, Nova Scotia, Canada; University of East Piedmont, Italy

## Abstract

**Background:**

On average, cognition declines with age but this average hides considerable variability, including the chance of improvement. Here, we investigate how exercise is associated with cognitive change and mortality in older people and, particularly, whether exercise might paradoxically increase the risk of dementia by allowing people to live longer.

**Methods and Principal Findings:**

In the Canadian Study of Health and Aging (CSHA), of 8403 people who had baseline cognition measured and exercise reported at CSHA-1, 2219 had died and 5376 were re-examined at CSHA-2. We used a parametric Markov chain model to estimate the probabilities of cognitive improvement, decline, and death, adjusted for age and education, from any cognitive state as measured by the Modified Mini-Mental State Examination. High exercisers (at least three times per week, at least as intense as walking, n = 3264) had more frequent stable or improved cognition (42.3%, 95% confidence interval: 40.6–44.0) over 5 years than did low/no exercisers (all other exercisers and non exercisers, n = 4331) (27.8% (95% CI 26.4–29.2)). The difference widened as baseline cognition worsened. The proportion whose cognition declined was higher amongst the high exercisers but was more similar between exercise groups (39.4% (95% CI 37.7–41.1) for high exercisers versus 34.8% (95% CI 33.4–36.2) otherwise). People who did not exercise were also more likely to die (37.5% (95% CI 36.0–39.0) versus 18.3% (95% CI 16.9–19.7)). Even so, exercise conferred its greatest mortality benefit to people with the highest baseline cognition.

**Conclusions:**

Exercise is strongly associated with improving cognition. As the majority of mortality benefit of exercise is at the highest level of cognition, and declines as cognition declines, the net effect of exercise should be to improve cognition at the population level, even with more people living longer.

## Introduction

As our understanding of dementia has evolved to imagine that its onset might be prevented, new attention is being paid to the role of physical activity as a potentially protective factor. People who report high levels of physical activity have a lower risk of dementia and cognitive decline than people who do not participate in physical activity. [1] With a single exception (a study that measured only exercise frequency but not intensity or duration [2]), many studies, using different measures of physical activity, in different populations, and with different follow up times have each concluded that people who participate in physical activity have a lower risk of dementia. [1] Clinical trials of up to 6 months support these findings, [3] in that people who participate in exercise programs demonstrate improved cognition. Animal studies suggest plausible causal mechanisms, including altered brain neurotrophic factors and decreased inflammation. [4]

Despite the demonstrated benefits of exercise on cognition, there is at least a theoretical concern that exercise might increase the burden of dementia. [5] Such a paradoxical result might occur if the mortality benefit of exercise were to trump the cognitive benefit. In other words, if more people lived to advanced ages as a result of exercising, and if the potency of age as the chief risk for dementia [6] was substantially undiminished by exercise, then the burden of dementia might increase with exercise even if the age-adjusted incidence is reduced. A definitive understanding of the interplay between exercise, cognition and mortality would require a long-term, randomized controlled trial. This is unlikely to happen, if only because there are too many health benefits associated with physical activity to ethically restrict exercise for any prolonged duration. While short-term controlled clinical studies can suggest that exercise improves cognition, [3] cognitive change is not just a short term phenomenon and cognitive decline occurs over many years. [7] In consequence, epidemiological studies are essential if we are to understand how cognitive change is associated with exercise over time.

Prior studies have examined the attenuation of decline in people who exercise using regression analyses.[8]–[14] However, the role of exercise on improvement, as opposed to attenuation of decline, has received relatively little study. To fully account for the complex dynamics of cognition,[10], [14]–[16], we use a multistate modeling approach to provide a unified description of cognitive change. As change is unlikely to be uniform across all cognitive states we estimate the chance of cognitive change in any direction and of any magnitude and of mortality for any given baseline cognitive score. We examined how exercise affects cognitive change, and whether differences between exercise groups reflects less decline, more frequent improvement, or differing mortality rates. To understand the complex interplay between exercise, mortality, and cognition, we evaluated these effects at all levels of baseline cognitive performance.

## Methods

**Figure 1:**
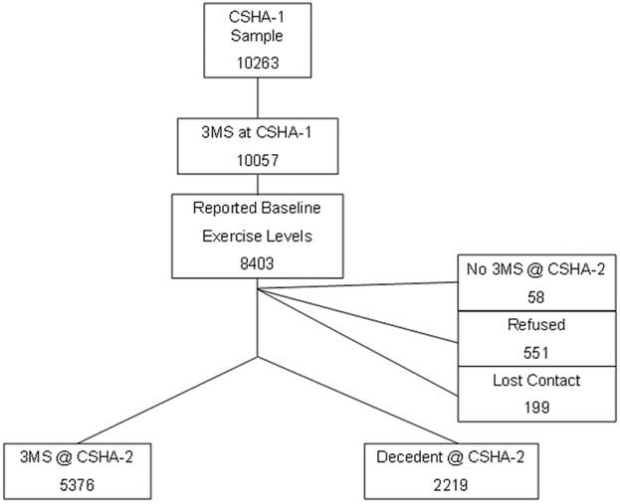
Selection of the study sample from the total CSHA-1 population, including those participants from CSHA-1 who completed the 3MS, reported exercise levels, and either completed the CSHA-2 3MS or died before follow up.

This is a secondary analysis of the Canadian Study of Health and Aging (CSHA), a national, multi-center, prospective cohort study of dementia in persons 65 years and older. In 1991, a representative population sample (N = 10 263) of people was drawn from provincial records as detailed elsewhere (Figure 1). [17] An initial interview screened for self-rated health, chronic conditions, functional ability, and cognition, the last using the Modified Mini-Mental State (3MS) examination. [18] In these analyses, we examined the change in cognition and risk of mortality at 5-year follow-up (CSHA-2), where the study consisted of the same components as at baseline (CSHA-1).

A self-administered risk factor questionnaire was completed at baseline and addressed demographic characteristics, occupational and environmental exposures, lifestyle, and medical and family histories. Two questions, based on the frequency and intensity of exercise, assessed the level of physical activity as validated elsewhere. [19] Here, we classified people as participating in ‘high exercise’ (three or more times per week, at least as intense as walking) and ‘low/no exercise’ (all other exercisers and non-exercisers). Of those people who completed the 3MS at CSHA-1 (n = 10 057), only participants who both answered the risk-factor questionnaire (n = 8403) and either completed a 3MS examination at CSHA-2 (n = 5376) or died between CSHA-1 and CSHA-2 (n = 2219) were included (Figure 1). In addition, people reported the number of years in formal education, which, with age, was entered as a covariate in the models.

### Cognitive States

As elaborated elsewhere, [20] cognitive states can be defined according to the number of errors in the Modified Mini-Mental State Examination (3MS). The 3MS [18] is a 100-point scale that extends the 30-point Mini Mental State Examination (MMSE) [21] by including tasks of animal naming, similarities, date and place of birth and a second recall task. For the complete 3MS questionnaire see http://www.csha.ca/r_community_questionnaire.asp These modifications have generally resulted in improved psychometric properties compared with the MMSE. [22], [23] Considering cognitive states in this way allows us to model transitions to better or worse cognitive states (or to dying) with more detail.[20] This approach to deficit accumulation (aggregated in a so-called ‘frailty index’) has previously allowed us to define frailty, and to observe characteristic behaviour of the frailty index. [24]–[28]

Successive cognitive states - from high cognition/low errors to impaired cognition/high errors - errors were grouped by 3's, where a 3-point difference on the 3MS is clinically detectable. [29] Thus, we consider that the “0” state is defined as 0, 1 and 2 errors (corresponding to 3MS scores = 100, 99 and 98). Likewise, the “1” state represents 3, 4 and 5 errors and so on until 3MS≤58 after which low numbers of people with those scores meant that they were combined as a 15^th^ state. Death was added as the 16^th^ state.

### Analysis

Differences (age, sex, education, hypertension, and exercise) were compared between high exercise and low exercise groups and between participants and non-participant groups using χ^2^ and Student's t-test as appropriate. In addition, the average 3MS change and the probability of cognitive stability/improvement, cognitive decline, and mortality were compared between high and low exercise groups. In order to fully understand the pattern of cognitive change, the probability of each cognitive outcome was examined both amongst survivors only and amongst all participants.

The frequencies of transitions between cognitive states were first aggregated in a transition matrix. The transition matrix gives a complete account of the proportion of people, from each baseline cognitive state, who transition to any cognitive state or death at follow up. A parametric Markov chain model was used to fit the empirical data to a modified Poisson distribution (Appendix S1). Goodness of fit was calculated by the coefficient of determination (R^2^) and the mean square error. To determine the effect of exercise and adjust for age and education in the model, the variables first were dichotomized (exercise, outlined above, age and education at the median, which is 76 years and 10 years respectively) and then incorporated in the model (Appendix S1).

The output of the model is four parameters that describe the probability of transitioning from any baseline cognitive state to any follow-up cognitive state or death between CSHA-1 to CSHA-2. Another four parameters describe the effect of exercise, age, and education on these transition probabilities. Note that the probabilities as determined by these parameters can be summed to display the predicted proportion of people who, from any cognitive state will improve or stay the same in cognition, worsen, or die. Furthermore, the relative risk of each of these outcomes for the low/no exercise group versus the high exercise group can be calculated by the ratio of these probabilities. (See the Appendix for the mathematical description of the model.)

## Results

Participants included in our analyses were younger, better educated, more likely to be male, and more often exercisers than non-participants. High-exercisers were younger, more often men, more educated, and less likely to be hypertensive than low/no-exercisers (Table 1).

**Table 1:** Descriptive traits of high versus low/no exercise groups and respondents versus non-respondents.

	High Exercise N = 3264	Low/No Exercise N = 4331	Cognitive Decline N = 2445	No Cognitive Decline N = 2931	Participants N = 7595	Non-participants N = 2668
Age (mean, sd)	74.4 (6·6)	77.4 (7·7)*	76.0 (6.7)	72.8 (5.9)†	76.1 (7.4)	77.7 (7.7) §
Sex (% female)	53.4%	64.4%*	59.6%	62.2%	60%	65%§
Education, yrs (mean, sd)	10.9 (3.9)	9.7 (3.7)*	10.1 (3.8)	10.9 (3.8)†	10.2 (3·8)	9.2 (4·0)§
Hypertension (% Yes)	32.1%	36.0%*	34.1%	33.8%	32.2%	34.3%
Exercise (% High)	100%	0%	44.6%	53.8%†	43·0%	34.2%§

* = significantly different between the high exercise and low exercise groups.

† = significantly different between participants with cognitive impairment and those with no cognitive impairment.

§ = significantly different between respondents and non-respondents.

On average, those people who participated in high levels of exercise showed less cognitive decline from baseline over 5 years (3.1 points on the 3MS) than did the low/no exercise group (5.5 points, p<0.001). Furthermore, among survivors, the high exercise group had less risk of cognitive decline (10.3% versus 15.8% in the low exercise group) and a higher chance of cognitive improvement/stability (89.7% versus 84.2% in low exercisers) (p<0.001).

**Figure 2:**
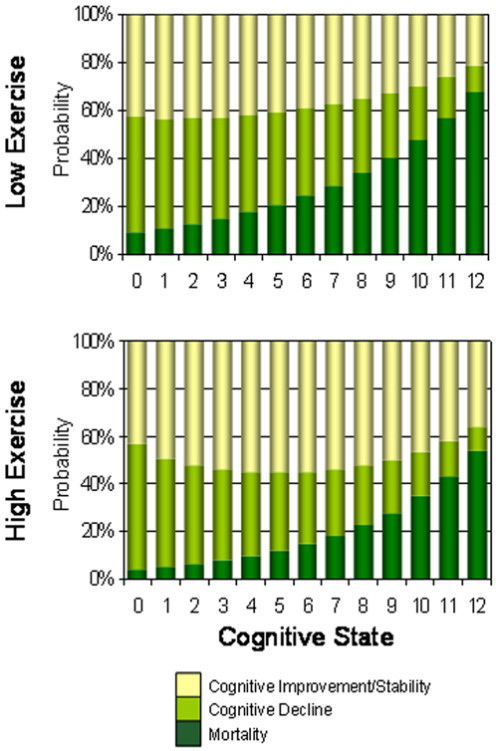
Changes in cognition in relation to baseline cognitive status at baseline by exercise level in unadjusted analyses.

When including all participants in the analyses, including non-survivors, the 3264 people who reported exercising at least as intensely as walking three or more times per week still experienced stable or improved cognition more often (42.3%, 95% CI 40.6–44.0) than did the 4331 who did not exercise this much (27.8%, 95% CI 26.4–29.2). The difference in improvement in relation to exercise was enhanced as baseline cognition worsened (Figure 2). However, because people in the high exercise group were also much less likely to die (18.3%; 95% CI 16.9–19.7 versus 37.5; 95% CI 36.0–39.0), not only did the high exercise group have more frequent stable or improved cognition but more high exercisers also declined in cognition (39.4%, 95% CI 37.7–41.1 compared with the low exercise group 34·8%; 95% CI 33·4–36·2). Even so, differences in the rates of cognitive decline between exercise groups were less than the differences in cognitive stability or improvement (Figure 2).

**Figure 3 pone-0003124-g003:**
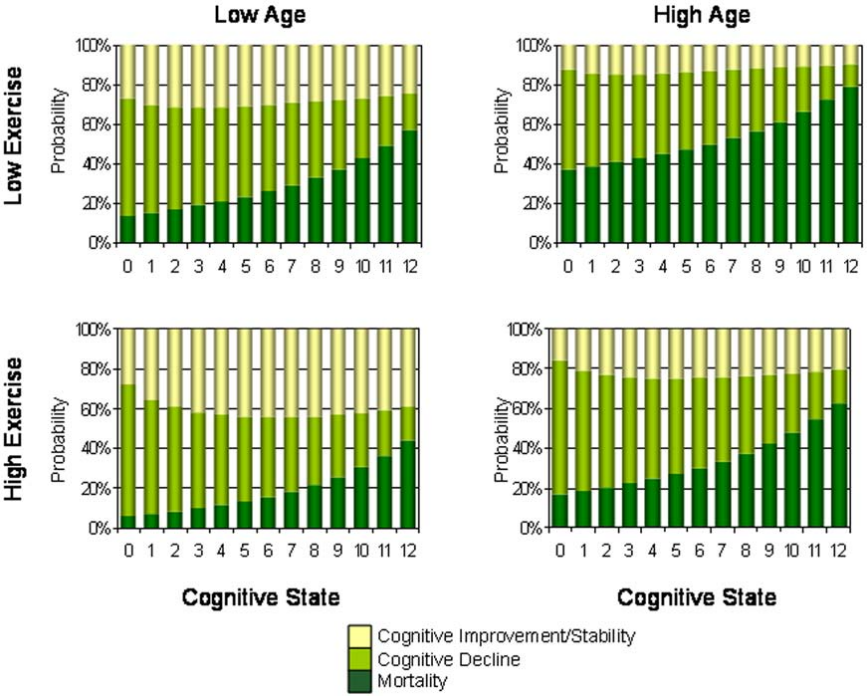
Changes in cognition in relation to baseline cognitive status at baseline by age and exercise level for people with lower levels of education.

**Figure 4:**
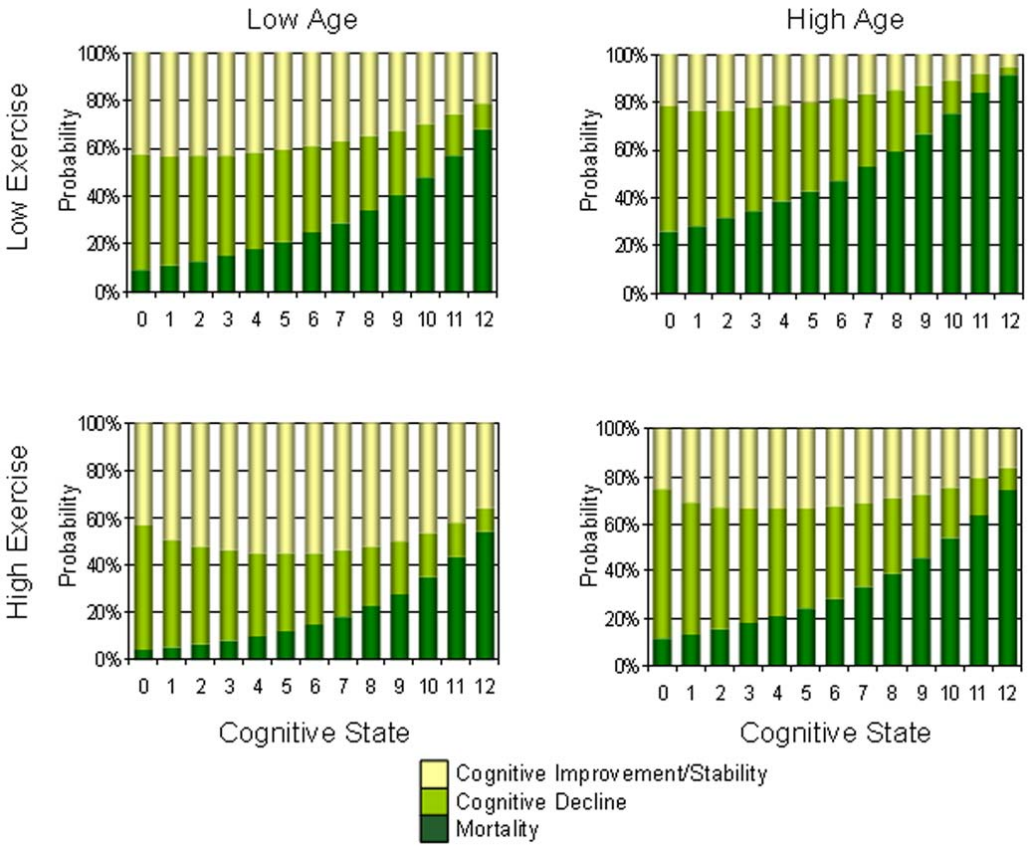
Changes in cognition in relation to cognitive status at baseline by age and exercise level for people with higher levels of education.

**Figure 5:**
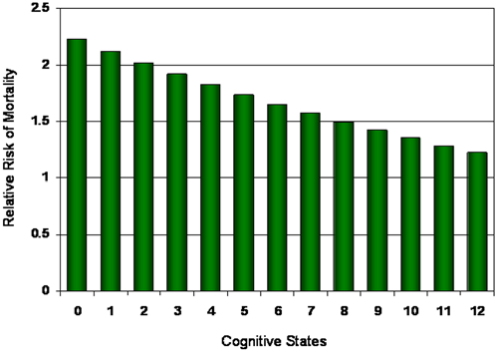
Relative risks of death for the low exercise group relative to the high exercise group by baseline cognitive state.

When taking into account age and education, the trends in cognitive transitions were similar to those in unadjusted analyses (Table 2). More people who exercised improved or stabilized in cognition at each baseline cognitive state, whether younger or older, more (Figure 3) or less educated (Figure 4). For all subgroups, the probability of death increased with worse cognition (Figures 3,4). The probability of death was also consistently less in the high exercise group than the low exercise group (Figures 2–4, Table 2). Note that while the absolute risk of death was always highest when cognition was lowest (Figures 2–4), the relative risk of low exercise on mortality was highest when cognition was highest (Figure 5). In short, the benefit of exercise to mortality is greatest in the best cognitive states.

**Table 2:** Average probabilities (and standard deviation) of getting better or maintaining the same cognitive performance, cognitive decline or dying and their standard deviations.

Age	Education	Probability of cognitive stability/improvement (Mean (SD))		Probability of cognitive decline (Mean (SD))		Probability of death (Mean (SD))	
		High Exercise	Low/No Exercise	High Exercise	Low/No Exercise	High Exercise	Low/No Exercise
Younger	Higher	0.49 (0.06)	0.36 (0.08)*	0.30 (0.12)	0.33 (0.12)	0.20 (0.16)	0.30 (0.18)
Younger	Lower	0.42 (0.06)	0.28 (0.03)*	0.39 (0.14)	0.28 (0.03)	0.23 (0.15)	0.34 (0.17)
Older	Higher	0.28 (0.06)	0·17 (0.06)	0.37 (0.16)	0.30 (0.16)	0.38 (0.19)	0.51 (0.19)
Older	Lower	0.22 (0.03)	0.12 (0.02)*	0.43 (0.16)	0·33 (0.13)	0.38 (0.17)	0.58 (0.16)

Older people are those whose age is at least 76 years old. Higher education means 10 years or more.

* Significant difference between exercise groups.

## Discussion

In this secondary analysis of the Canadian Study of Health and Aging, we evaluated the impact of exercise on cognitive change in elderly people over five years. By mapping the transitions in cognitive states in relation to exercise, and adjusting for age and education, we found that people who participated in high levels of physical activity had a greater chance of stabilizing or improving their cognition compared with people who participated in low/no exercise, especially as baseline cognition worsened. Interestingly, the chance of cognitive decline was more similar between exercise groups. High exercise was also associated with a lower risk of death but this effect was greatest when cognition was highest, and was attenuated as cognition worsened.

Our data must be interpreted with caution. The follow-up period was only 5 years and the effects of exercise on cognitive transitions need to be examined for longer follow-up periods. Although the CSHA is a large, representative sample, 9.6% were lost to follow up. Those lost to follow-up were younger, more often female, less educated, and less likely to exercise than participants.. These differences may have affected the association between exercise and cognition. Misreporting of physical activity is also of concern. However, by using broad groups of activity levels, we minimize the impact of this bias. Misclassification between exercise groups is possible, but unlikely to have been systematic in relation to cognitive testing in that the self-assessed risk factor questionnaire, where the exercise/exposure data were collected, was completed independently of the 3MS.

Because our model is new, the results must be evaluated with care, and skepticism is likely. Even so, the model is well grounded in several earlier papers regarding cognition and frailty. [24]–[28] Here, we have included adjustment for three covariates, which allowed us to compare risks between groups of exercisers and adjust for two confounders, age and education. By using the output of the model to calculate probabilities of cognitive events, we were better able to describe the dynamic changes of the brain that occur with ageing. [15], [16] Furthermore, we were able to present a unified model of relationship between exercise and cognition, taking into account baseline cognition, exercise levels, and mortality. Nevertheless, the model is not perfect. Because the fit of the model to the cognitive transitions is sacrificed as more covariates are incorporated, we only adjusted for two confounders (age and education). As a result, this data should not be interpreted in isolation but in light of the past and future studies of different designs, in diverse populations, controlling for numerous confounders which have concluded that exercise is independently associated with cognitive performance.

According to both previous epidemiological studies using the CSHA sample [30] and those of other groups, [1] exercise is associated with a reduced risk of cognitive decline. Additionally, clinical trials [3] have suggested that people who exercise can have improved cognition. The difference between an improved chance of cognitive improvement and a reduced chance of cognitive decline is subtle. When only survivors are included, as was the case in previous regression analyses, [31]–[33] the probability of stability or improvement is the complement to the probability of cognitive decline. However, when considering three or more possible outcomes, cognitive stability/improvement, cognitive decline, and mortality, this is not the case. Not all cognitive outcomes need be altered to the same degree. Here, cognitive decline, slightly higher in risk but lower in magnitude in high exercisers, was present in about the same proportion across exercise groups, By contrast, the chance of stability/improvement was much higher amongst people who exercise. Optimistically, this suggests that even people with cognitive impairment may be able to improve their cognition by starting an exercise program. It also reinforces the contention that, rather than viewing changes with age as a picture of gradual loss, a more dynamic understanding of what is happening in the older brain needs to be considered.[16] Such a consideration might well explore practical means of not just attenuating decline, but of augmenting improvement, as is illustrated here for exercise.

It is possible that the association between exercise and cognition is not a causal relationship. Low exercise at baseline might reflect early cognitive impairment; this has been a criticism of papers that have evaluated exercise in relation to diagnostic states.[34]–[36] In reply to such criticism, the relationship has been found to hold when other potential confounders – such as vascular risk or functional impairment – were included in the adjustment. The evaluation of cognitive states is likely less susceptible to problems in the direction of the association, i.e. given that transitions were considered from each cognitive state and because each cognitive state only included a 3 point range of scores on the 3MS, the variation in cognition at baseline between exercise groups in each cognitive state is unlikely to show such a tight gradient. Even if it were confounded, the closely graded nature of the link – i.e. that people with a shade more cognitive errors were a shade less likely to exercise - would be of interest.

Since exercise is associated with increased longevity, [37] there is recurring concern that exercise may delay cognitive impairment but still increase the duration of impairment, giving the paradoxical result of more cognitive impairment on a population basis.[5] Our study provides some evidence against this paradox being true. The analyses suggest that most of the extension of life occurs in the least impaired states, so that exercise may extend longevity without prolonging time in impaired states.

Our analyses better quantify the nature of the apparent benefit conferred by exercise, and draw attention to the possibility of improvement in cognition with age. The analyses suggest too that most of the extended life occurs in the least impaired states. That the increased likelihood of improving was consistent for all stages of cognitive decline suggests that it is neither age nor cognitive impairment alone are reasons not to exercise.

## Supporting Information

Appendix S1 (0.04 MB DOC)

Figure S1 Schematic representation of the chain transitions between the different cognitive states (S_i_, i = 0,1,2,3,…) and death (D). Here, error groups are represented by the number of errors on the 3MS in groups of 3 (e.g. 0–2 errors = S_0_, 3–5 errors = S_1_, etc). By follow-up, that person can have the same error group, or transit to a new group, represented by fewer or more errors, or can die.
(0.04 MB TIF)

Figure S2 The probability of transition from cognitive error group n to group k (the first 9 groups are presented). Error groups are defined by errors on the 3MS in 3 point groupings intervals (states) (i.e. state 0 is 0–2 errors, state 1 is 3–5 errors, etc.). The blue circles represent observational data for 5-year transitions for high-exercisers and red circles represent data for low/no-exercisers. The blue and red lines represent the model fit for high- exercisers and low/no-exercisers respectively.
(0.07 MB TIF)

Figure S3 The probability of death as a function of cognitive error at baseline. The blue circles represent observational data for 5-year transitions for high-exercisers and red circles represent data for low/no-exercisers. The blue and red lines represent the model fit for high- exercisers and low/no-exercisers respectively. The goodness of fit shown by the correlation coefficients between observational frequencies and the model fit (Equations S3) for each exercise group.
(0.03 MB TIF)

Table S1 Parameter estimates the Poisson Model fit to transitions in 3MS error states and their 95% confidence intervals.
(0.05 MB DOC)
