# Supplementary material for: Changes in Cognition and Mortality in Relation to Exercise in Late Life: A Population Based Study
Source: PLoS One. 2008 Sep 1;3(9):e3124. doi: 10.1371/journal.pone.0003124 (PMC2518854; doi:10.1371/journal.pone.0003124)
Supplement: Appendix S1 — (0.04 MB DOC) [file pone.0003124.s001.doc]

**APPENDIX S1**

*The model*

We used the following stochastic model to describe changes in individual cognitive status as a Markov chain. Figure S1 illustrates the general schema of transitions between the different states (*S*_i_, *i* = 0,1,…) including death (D). This model builds on our earlier work,[S1-S3] except that instead of considering health deficits, here we consider as deficits cognitive status in relation to the number of errors made on the 3MS.[S4] With any individual’s initial cognitive state as ‘*n*’, let $P_{nk}$ be the probability that this individual will have cognitive state ‘*k*’ at the time of the next assessment, and let $P_{nd}$ be the probability of dying before the next assessment (i.e., the absorbing state). We have shown that the transition probabilities between the different numbers of states can be approximated as:

$P_{nk}\approx\frac{\rho_{n}^{k}}{k!}\exp(-\rho_{n})(1-P_{nd})$ (S1)

Strictly speaking, this is an approximation, because not all the transition states can be modeled informatively – i.e. we cannot model an infinite number of transitions, which would be required for a Poisson distribution. In consequence, we must observe a finite number of states. Here, the maximum number of states to be observed (other than the absorbing state) is 34, i.e. 100/3, which is less than infinity. (Note too that empirically, 99% of all observations are seen in the first 20 transitions and 98% for the first 16 states.) Even so, the model accounts for all outcomes observed here, i.e. sum of the probability of transiting from each state to every other transient (non-absorbing) state is the survival probability; in other words, these probabilities sum to 1-*P_nd_*.

As the term 1-*P_nd_* is the probability of survival between two assessments, for each *n*, the transition probabilities satisfy a modified (by accounting for the survival probability) Poisson distribution in which the parameter *ρ * depends on the current state *n* as follows:

*ρ*_n_ = *a_1_ +b*_1_ *n* (S2)

This parameter can increase with *n* differently with age, sex and other factors. The probability of death can be parameterized in many ways; consider here the following approximation:

*P_nd_* = exp(*a*_2_ +*b*_2_ *n*) (S3)

The interpretation of the parameters is straightforward: *a*_1_ is *ρ*_0_ (it is the mean follow up cognitive state (*k*) given *zero* state at baseline, i.e. *n*=0) and *a*_2_ is the (negative) logarithm of the probability of survival at zero state. The zero-state parameters *a*_1_ and *a*_2_ are estimates of the ambient probabilities of death and of accumulating deficits respectively. The *b*_1_ and *b*_2_ are the state increments when *n*>0. Similar to the Poisson parameter, the probability of death can increase with *n* differently with age sex and other covariates. To incorporate the covariates we consider that each of 4 parameters can be represented as a linear function of *m* covariates *z*_i_ (*i* =1,…*m*)

$a_{j}=\alpha_{j}+\sum_{i=1}^{m} \gamma_{i}^{j}z_{i}$ (S4a)

$b_{j}=\beta_{j}+\sum_{i=1}^{m} \delta_{i}^{j}z_{i}$ (S4b)

where *j*=1,2 for transitions between the cognitive states and from cognitive states to death, respectively. In this notation, the regression coefficients *gammas* modify the estimates of *a_j_* and *deltas* modify the estimates of *b_j_.* The model is represented by equations (S1) - (S4).

The parameters of the model and their confidence intervals were estimated using a nonlinear least squares procedure in Matlab (version 7.5). The codes are available for academic use upon request.

The output of the model shows all possible outcomes (improving, staying the same, worsening or dying). Figure S2 shows that these conform to a Poisson distribution. As expected, the distribution of cognitive states is shifted to the right (worse cognition) for each progressive baseline cognitive states. As expected, people with worse baseline cognition also have worse follow up cognition. Furthermore, for each successive state, the area under the curve is less, meaning that fewer people survived. Figure S3 shows that the probability of death increases exponentially (Equation S3) with worse baseline cognition in both exercise groups.

Appendix S1: References

1. Mitnitski A, Bao L, Rockwood K (2006). Going from bad to worse: a stochastic model of transitions in deficit accumulation, in relation to mortality. Mech Ageing 127(5): 490-3.
2. Mitnitski A, Song X, Rockwood K (2007). Improvement and decline in health status from late middle age: modeling age-related changes in deficit accumulation. Exp Gerontol 42(11): 1109-15.
3. Rockwood K, Mitnitski A (2007). Frailty, fitness and the mathematics of deficit accumulation of deficits. Rev Clin Gerontol 17: 1-12.
4. Mitnitski A, Rockwood K (2008). Transitions in cognitive test scores over 5 and 10 years in elderly people: evidence for a model of age-related deficit accumulation. BMC Geriatrics 8: 3.
