# Supplementary figures and images for: Changes in Cognition and Mortality in Relation to Exercise in Late Life: A Population Based Study
Source: PLoS One. 2008 Sep 1;3(9):e3124. doi: 10.1371/journal.pone.0003124 (PMC2518854; doi:10.1371/journal.pone.0003124)

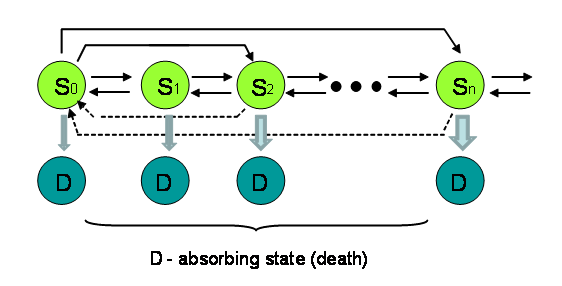

Supplement: Figure S1 — Schematic representation of the chain transitions between the different cognitive states (Si, i = 0,1,2,3,…) and death (D). Here, error groups are represented by the number of errors on the 3MS in groups of 3 (e.g. 0–2 errors = S0, 3–5 errors = S1, etc). By follow-up, that person can have the same error group, or transit to a new group, represented by fewer or more errors, or can die. (0.04 MB TIF) [file pone.0003124.s002.tif]

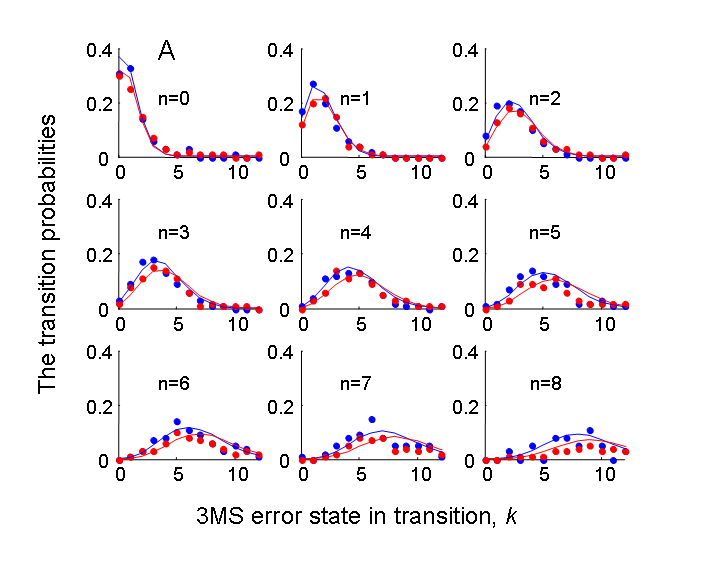

Supplement: Figure S2 — The probability of transition from cognitive error group n to group k (the first 9 groups are presented). Error groups are defined by errors on the 3MS in 3 point groupings intervals (states) (i.e. state 0 is 0–2 errors, state 1 is 3–5 errors, etc.). The blue circles represent observational data for 5-year transitions for high-exercisers and red circles represent data for low/no-exercisers. The blue and red lines represent the model fit for high- exercisers and low/no-exercisers respectively. (0.07 MB TIF) [file pone.0003124.s003.tif]

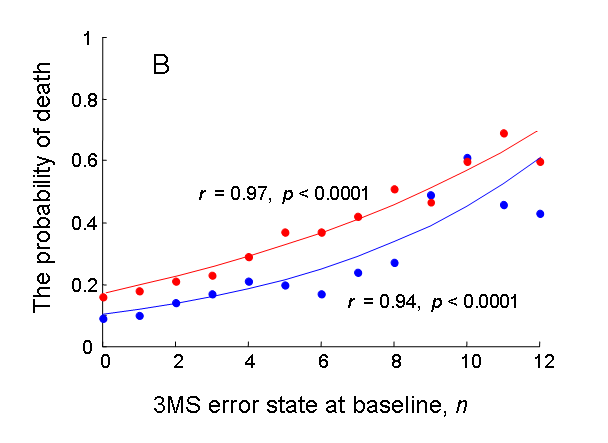

Supplement: Figure S3 — The probability of death as a function of cognitive error at baseline. The blue circles represent observational data for 5-year transitions for high-exercisers and red circles represent data for low/no-exercisers. The blue and red lines represent the model fit for high- exercisers and low/no-exercisers respectively. The goodness of fit shown by the correlation coefficients between observational frequencies and the model fit (Equations S3) for each exercise group. (0.03 MB TIF) [file pone.0003124.s004.tif]
