# Supplementary material for: Changes in Cognition and Mortality in Relation to Exercise in Late Life: A Population Based Study
Source: PLoS One. 2008 Sep 1;3(9):e3124. doi: 10.1371/journal.pone.0003124 (PMC2518854; doi:10.1371/journal.pone.0003124)
Supplement: Table S1 — Parameter estimates the Poisson Model fit to transitions in 3MS error states and their 95% confidence intervals. (0.05 MB DOC) [file pone.0003124.s005.doc]

Table S1. Parameter estimates the Poisson Model fit to transitions in 3MS error states and their 95% confidence intervals.

| Covariate | Parameter | Model 1  Adjusted for Exercise | Model 2  Adjusted for Age | Model 3  Adjusted for Education | Model 4  Adjusted for Exercise, Age, Education |
| --- | --- | --- | --- | --- | --- |
|  | α_1_ | 0.88 (0.70, 1.06) | 0.85 (0.68, 1.01) | 0.87 (0.71, 1.03) | 0.79 (0.54, 1.05) |
|  | β_1_ | 0.94 (0.86, 1.02) | 0.89 (0.83, 0.96) | 0.95 (0.87,1.03) | 0.83 (0.72, 0.93) |
|  | α_2_ | -2.28 (-2.48,-2.07) | -2.32 (-2.55,-2.10) | -1.84 (-1.97,-1.72) | -3.13 (-3.49,-2.76) |
|  | β_2_ | 0.15 (0.13,0.17) | 0.13 (0.11,0.15) | 0.13 (0.12,0.14) | 0.21 (0.18,0.24) |
| Exercise | γ_1_^1^ | 0.02 (-0.25, 0.30) |  |  | -0.04 (-0.35, 0.27) |
|  | δ_1_^1^ | 0.14 (0.01, 0.27)* |  |  | 0.14 (0.01, 0.26)* |
|  | γ_1_^2^ | 0.69 (0.45, 0.93)* |  |  | 0.80 (0.53, 1.08)* |
|  | δ_1_^2^ | -0.05 (-0.07, -0.03)* |  |  | -0.05 (-0.07, -0.03)* |
| Age | γ_2_^1^ |  | 0.46 (0.13, 0.78)* |  | 0.46 (0.12, 0.80)* |
|  | δ_2_^1^ |  | 0.18 (0.05, 0.32)* |  | 0.13 (-0.01, 0.26) |
|  | γ_2_^2^ |  | 0.87 (0.62, 1.13)* |  | 0.98 (0.69, 1.27)* |
|  | δ_2_^2^ |  | -0.04 (-0.07, -0.02)* |  | -0.06 (-0.08, -0.03)* |
| Education | γ_3_^1^ |  |  | 0.53 (0.24, 0.81)* | 0.41 (0.09, 0.73)* |
|  | δ_3_^1^ |  |  | 0.01 (-0.11, 0.13) | 0.05 (-0.07, 0.18) |
|  | γ_3_^2^ |  |  | 0.02 (-0.17, 0.20* | 0.36 (0.08, 0.64)* |
|  | δ_3_^2^ |  |  | -0.02 (-0.03, 0.00) | -0.05 (-0.08, -0.03)* |
| R |  | 0.930 | 0.928 | 0.952 | 0.815 |
| R^2^ |  | 0.865 | 0.861 | 0.905 | 0.665 |
| Mean square error |  | 0.0019 | 0.0018 | 0.0014 | 0.0058 |

* - statistically insignificant difference between covariate groups (p>0.05)

α_1_ and α_2_ describe the background risk of cognitive errors and mortality respectively. β_1_ and β_2_ describe the increased risk of cognitive errors and mortality associated with each additional baseline cognitive error. In addition, the effect of γ_i_^1^, δ_j_^1^, γ_i_^2^, δ_j_^2^ show the effect of exercise, age and education on α_1_, β_1_, α_2_, and β_2_ respectively. The goodness of fit (R^2^, mean square error) of the modified Poisson distribution is displayed.
